# The thrifty phenotype hypothesis revisited

**DOI:** 10.1007/s00125-012-2589-y

**Published:** 2012-05-30

**Authors:** A. A. Vaag, L. G. Grunnet, G. P. Arora, C. Brøns

**Affiliations:** 1 Department of Endocrinology, Rigshospitalet (7652), Blegdamsvej 9, 2100 Copenhagen Ø, Denmark; 2 Copenhagen University, Copenhagen, Denmark; 3 Lund University, University Hospital Malmö, Malmö, Sweden; 4 Deep Hospital, Ludhiana, India

**Keywords:** Fetal programming, Intergenerational prevention, Metabolic syndrome, Thrifty phenotype, Type 2 diabetes

## Abstract

Twenty years ago, Hales and Barker along with their co-workers published some of their pioneering papers proposing the ‘thrifty phenotype hypothesis’ in *Diabetologia* (4;35:595–601 and 3;36:62–67). Their postulate that fetal programming could represent an important player in the origin of type 2 diabetes, the metabolic syndrome and cardiovascular disease (CVD) was met with great scepticism.

More recently, their observations have been confirmed and expanded in many epidemiological and animal experimental studies, and human integrative physiological studies have provided insights into some of the underlying molecular mechanisms. Type 2 diabetes is a multiple-organ disease, and developmental programming, with its idea of organ plasticity, is a plausible hypothesis for a common basis for the widespread organ dysfunctions in type 2 diabetes and the metabolic syndrome. Only two among the 45 known type 2 diabetes susceptibility genes are associated with low birthweight, indicating that the association between low birthweight and type 2 diabetes is mainly non-genetic. Prevention programmes targeting adult lifestyle factors seems unable to stop the global propagation of type 2 diabetes, and intensive glucose control is inadequate to reduce the excess CVD mortality in type 2 diabetic patients. Today, the thrifty phenotype hypothesis has been established as a promising conceptual framework for a more sustainable intergenerational prevention of type 2 diabetes.

Research into insulin resistance, type 2 diabetes and the metabolic syndrome took off in the 1960s after the first human plasma insulin assays demonstrated that the majority of cases of late-onset diabetes could not be explained sufficiently by lack of insulin [1]. Twin studies from the 1980s suggested an almost exclusive role of genetics in type 2 diabetes, with nearly 100% concordance rates among genetically identical monozygotic twins [2]. The studies of Hales and Barker et al. [3, 4], published in *Diabetologia* in the early 1990s, were therefore highly provocative to the diabetes research community, postulating that not only type 2 diabetes, but also the key components of the metabolic syndrome, representing well-established cardiovascular disease (CVD) risk factors, seemed to have at least parts of their origin in early life. The notion that factors operating early in life influence the risk of type 2 diabetes and CVD decades later was originally devised by the Norwegian epidemiologist Anders Forsdahl in the 1970s [5]. Nevertheless, the undisputable achievement of Hales and Barker was that, in an unselected population sample from Hertfordshire, UK, they proved a direct link between low weight at birth and increased risks of developing type 2 diabetes, hypertension, elevated triacylglycerols and insulin resistance later in life [3, 6]. Thus, the paradigm-shifting potential of the studies was not only the idea of a role of fetal programming in itself but, equally as important, the notion that fetal programming could represent a significant player in the origin of type 2 diabetes, the metabolic syndrome and CVD.

Criticism of the studies of Hales and Barker included the use of changing definitions of markers of growth in early life and underdevelopment in different studies, such as birthweight versus ponderal index with respect to exposure, and different phenotypes such as type 2 diabetes, the metabolic syndrome and insulin resistance with respect to outcome. As disbelievers ignored the provocative ideas, supporters gathered in societies somewhat isolated from the general community of diabetes research. This polarisation might have impeded the speed at which the ideas of fetal programming were widely accepted. For example, technological developments within molecular biology, such as the genome-wide association platform, were more rapidly adopted by researchers in the field of type 2 diabetes genetics, which facilitated the identification of a fascinating scenario of distinct molecular causes and disease mechanisms of type 2 diabetes [7]. These important achievements must be considered in the context of the fact that, although more than 45 genes are currently documented to be associated with type 2 diabetes, these quantitatively account for only around 10% of the *primary constitutional* origin of type 2 diabetes [7]. Accordingly, the major primary aetiological factors involved in type 2 diabetes remain unexplained, which leaves substantial potential for early life determinants to be central in the aetiology of type 2 diabetes.

Alongside the diverse beliefs and conceptions regarding the aetiology of type 2 diabetes and the metabolic syndrome, there was a strong polarisation of views in the 1980s and 1990s concerning the roles of muscle insulin resistance, defective pancreatic insulin secretion and elevated hepatic glucose production in the pathophysiology of type 2 diabetes. This debate has now been settled by a uniform agreement that glucose intolerance, ranging from the prediabetic states of impaired fasting glucose and impaired glucose tolerance to overt type 2 diabetes, constitutes heterogeneous dysmetabolic states, involving the dysfunction of multiple organs, including the liver, muscle, pancreas, adipose tissue, gut, kidney and brain [8]. It is of interest that the concept of fetal programming, with its ideas of organ plasticity [4], may represent the most plausible hypothesis of a common ground for the underlying aetiology and molecular mechanisms of type 2 diabetes. Thus, the multiple organ dysfunctions in type 2 diabetes, changing with time and age, and differing in magnitude between type 2 diabetic patients within and between societies, require a comprehensive conceptual framework such as developmental programming, as illustrated in Fig. 1. Type 2 diabetes genes have appeared to be more selective in their influence on organ dysfunctions, with defective pancreatic insulin secretion being the most important [7]. In contrast, despite their initial hypothesis of fetal programming of type 2 diabetes being associated with impaired insulin secretion [4], Hales and Barker and colleagues were the first to document the association between low birthweight and insulin resistance in humans [6]. The importance of this finding is underscored by our knowledge that insulin resistance is a very prominent and early feature of the metabolic syndrome and has consistently been associated with fetal programming in humans, regardless of whether the exposure is low birthweight, prematurity independent of low birthweight, gestational diabetes mellitus (GDM) or twin/zygosity status [9–11].

**Fig. 1:**
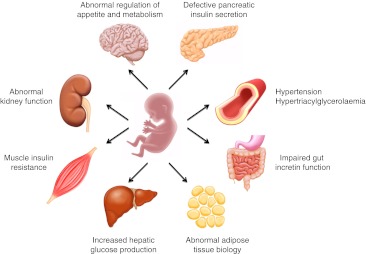
The developmental origin of type 2 diabetes provides a conceptual framework to explain the multiple organ defects in type 2 diabetes

Regarding the definition of the metabolic syndrome, central points in the discussion of its raison d´être over recent years have been whether it is a real disease entity of clinical relevance, if insulin resistance is the main common denominator or cause, if it explains the risk of CVD beyond the contributions of each of the individual components, and whether the clustering of the proposed components of the metabolic syndrome may be an artefact when observed from a stringent epidemiological perspective. What has not been sufficiently emphasised in this debate is that the thrifty phenotype hypothesis, formulated with great visionary precision by Hales and Barker et al [3], represents the most plausible explanation of the origin of all of the components of the metabolic syndrome.

The idea of an adverse fetal environment rather than genetic determinants defining the origin of type 2 diabetes, the metabolic syndrome and CVD was supported by twin studies reporting lower birthweights among monozygotic twins with type 2 diabetes compared with their genetically identical but non-diabetic co-twins [12]. Subsequent twin studies documented that both defective insulin secretion and insulin resistance were associated with low birthweight in a complex non-genetic and age-dependent manner [11]. The age dependency may in this context represent an important issue to explain the highly age-dependent states of type 2 diabetes and the metabolic syndrome, as well as CVD. While the concordance estimates from population-based twin studies questioned the notion of a major genetic component in type 2 diabetes [13], perhaps the most significant lesson from twin studies was the finding that zygosity—and thus twin status—may influence insulin secretion and insulin action [11]. The extent to which twins develop type 2 diabetes in a differential age-dependent manner compared with singletons is currently unresolved [13, 14], but the finding that twins have a risk of type 2 diabetes that is similar to a singleton with a lower birthweight is consistent with the notion that twins may differ from singletons with respect to risk of type 2 diabetes. Thus, the thrifty phenotype hypothesis has challenged the role of the classic twin model in the assessment of genetic versus non-genetic risk of metabolic and CVD diseases, emphasising the enormous impact of the hypothesis.

Despite low birthweight being a crude marker of an adverse intrauterine environment, it has, with remarkable consistency, been associated with risk of type 2 diabetes as well as impaired insulin secretion and insulin resistance in multiple studies, including population-based epidemiological investigations [10]. In addition, studies of the identified genetic type 2 diabetes markers have confirmed the notion that the association is predominantly non-genetic. While prematurity is associated with risk of type 2 diabetes independently of birthweight [10], studies of third trimester fetal growth rate pointed towards effects prior to or after the third trimester (i.e. all periods except the third trimester) being the most important periods during development relevant to programming of components of the metabolic syndrome [15].

Animal studies have provided substantial ‘proof of concept’ for associations of global as well as protein undernutrition during pregnancy with glucose intolerance and physiological and metabolic defects relevant to type 2 diabetes in their offspring [16]. Importantly, comparative studies of low birthweight in humans and protein-undernourished rats in utero identified strikingly similar changes in the expression of key insulin signalling proteins and of the glucose transporter GLUT4 in both skeletal muscle and adipose tissue [17]. The increasing awareness of a prime role of epigenetics, including DNA methylations and histone modifications, as well as regulatory microRNAs, has provided ideas and novel tools to look for the mechanisms that underlie the developmental programming of organ defects relevant to insulin resistance and type 2 diabetes, forecasting groundbreaking discoveries within the years to come. Prominent examples of recent discoveries in the area include transcriptional regulation by promoter DNA methylation and histone modifications of the key pancreatic proliferation and transcription factor Pdx1 by fetal undernutrition [16], and the discovery of increased expression of miR-483-3p in subcutaneous adipose tissue from humans with a low birthweight and from rats that were protein undernourished in utero, conferring an increased risk of lipotoxicity with its detrimental effects on the functioning of organs associated with type 2 diabetes [18].

The extent to which the global diabetes epidemic may be driven by a mismatch between being born with a low birthweight and the fast propagation of overnutrition and physical inactivity seen over recent years in developing countries needs to be determined to provide a focus for efforts to prevent metabolic diseases. GDM may be considered an early manifestation of type 2 diabetes that is unmasked by pregnancy-induced insulin resistance, and studies in both animals and humans have indicated that exposure to an adverse fetal environment is a significant risk factor for GDM per se [19]. Besides compelling evidence of intergenerational transmission of type 2 diabetes and the metabolic syndrome via low birthweight and GDM, recognition of this has provided support for universal GDM screening, and for pregnancy being a window of opportunity to prevent type 2 diabetes in both mother and child. The relevance and importance of earlier and more effective prevention of type 2 diabetes and the increased CVD mortality associated with this condition is underscored by the modest effects of early detection (and implementation of treatment) by screening for type 2 diabetes in the general population on type 2 diabetes-associated mortality [20], as well as the absence of any significant effect of intensive as opposed to conventional glucose control on CVD mortality in patients with overt type 2 diabetes [21]. To understand the full potential of the thrifty phenotype hypothesis as a platform to implement primary prevention of type 2 diabetes there is an urgent need to determine the extent to which developmental programming influences the development of type 2 diabetes in different populations and to understand the long-term effects of exposures during pregnancy and the distinct molecular mechanisms involved.

## Abbreviations

CVD: Cardiovascular disease
GDM: Gestational diabetes mellitus
